# Bilateral Orbital Cavernous Hemangiomas

**Published:** 2010-01

**Authors:** Maryam Aletaha, Reza Erfanian Salim, Abbas Bagheri, Hossein Salour, Mohammad Abrishami

**Affiliations:** Labbafinejad Medical Center, Shahid Beheshti University of Medical Sciences, Tehran, Iran

A 42-year-old woman presented with loss of vision and progressive proptosis in her right eye from one year before. Best-corrected visual acuity was 20/30 and 20/20 in her right and left eyes, respectively. A mild relative afferent pupillary defect existed on the right side. Exophthalmometric readings were 27 and 20 mm in the right and left eyes, respectively with no ocular deviation or restriction in motility. Both eyes had unremarkable anterior segments and normal intraocular pressure. Funduscopy revealed right optic disc swelling. Automated perimetry (central 24 - 2 threshold test) demonstrated an enlarged blind spot together with generalized depression in the right eye (Fig. 1) but was normal on the left side. General physical examination, laboratory tests and chest radiography were within normal limits.

Orbital computed tomography (CT) scanning showed a round and well-defined homogenous intraconal soft tissue mass, 22 mm in diameter causing severe proptosis in the right orbit and a similar lesion 11 mm in diameter in the superomedial extraconal space in the left orbit (Fig. 2). Orbital magnetic resonance imaging (MRI) revealed the lesions to be hypointense relative to fat on T_1_-weighted and isointense relative to muscle on T_2_-weighted images with marked uniform enhancement after gadolinium injection (Fig. 3). Imaging findings were compatible with bilateral orbital cavernous hemangiomas.

The patient underwent orbital surgery on the right side using a sub-brow Wright incision and lateral wall orbitotomy, and a large encapsulated purplish soft mass was excised. One month later orbital surgery using a Lynch incision was performed on the left side and a similar but smaller mass was excised. Histopathologic reports for both lesions were similar: well-defined proliferation of multiple dilated vascular structures with a single layer of endothelial cells, containing blood and surrounded by inflammatory fibroconnective tissue (Fig. 4). These findings were compatible with cavernous hemangioma.

## DISCUSSION

Cavernous hemangioma is the most common benign orbital mass lesion in adults and is more common in female subjects in the second to fifth decades of life.^1^ It is presumed to be a low-flow vascular malformation or hamartoma present at birth which undergoes enlargement later in life.^2^ Painless, gradually progressive proptosis and visual disturbance are common clinical signs,^3^ but cases of rapid progression^4^ and severe restriction in motility^5^ have also been reported.

Orbital cavernous hemangioma is believed to be almost always unilateral, but few cases of bilateral involvement have been reported.^6^–^9^ Two reports of bilateral lesions also exist in the literature, one as a part of Maffucci’s syndrome;^2^ the other as bilateral multifocal hemangiomas associated with the blue rubber bleb nevus syndrome.^10^ Paonessa et al^11^ evaluated MRI characteristics of 14 patients with surgically confirmed orbital cavernous hemangiomas and found 3 subjects with bilateral involvement. They concluded that technological advances and closer attention to the contralateral orbit, may lead to detection of more cases of bilateral involvement. As evident in the patient presented herein and other similar reports, ophthalmologists should keep in mind that bilateral orbital cavernous hemangiomas may be more common than initially believed.

## Figures and Tables

**Figure 1:**
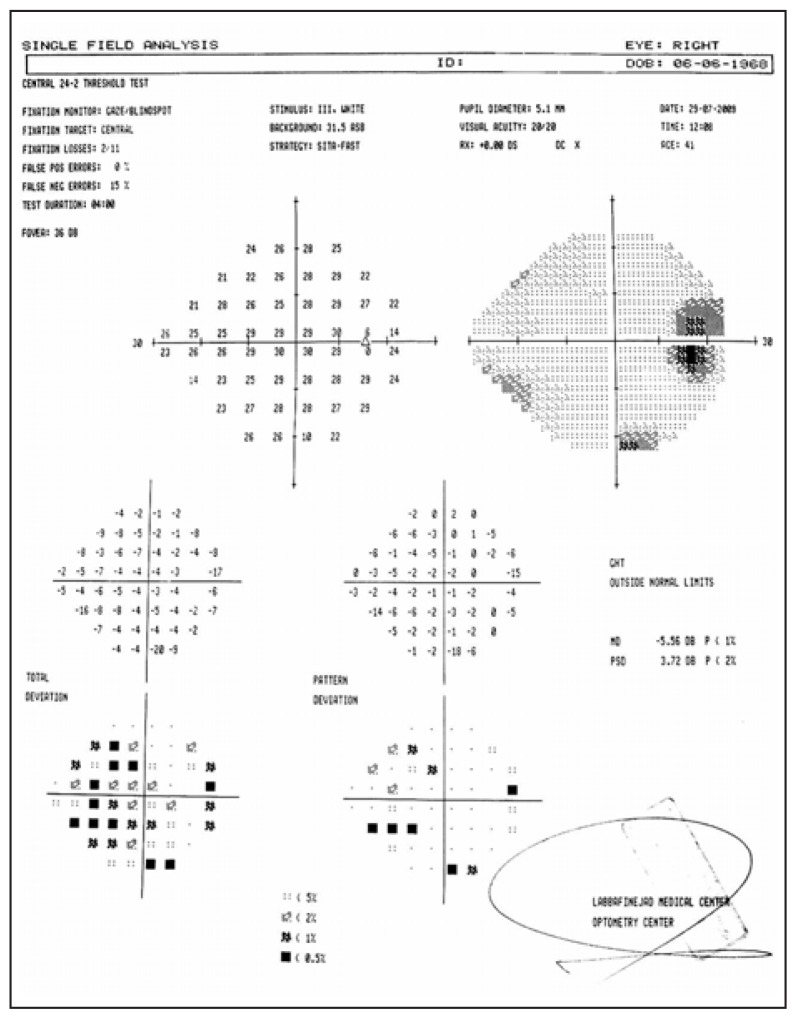
Automated perimetry (central 24-2 threshold test) shows an enlarged blind spot with generalized depression in the right eye.

**Figure 2:**
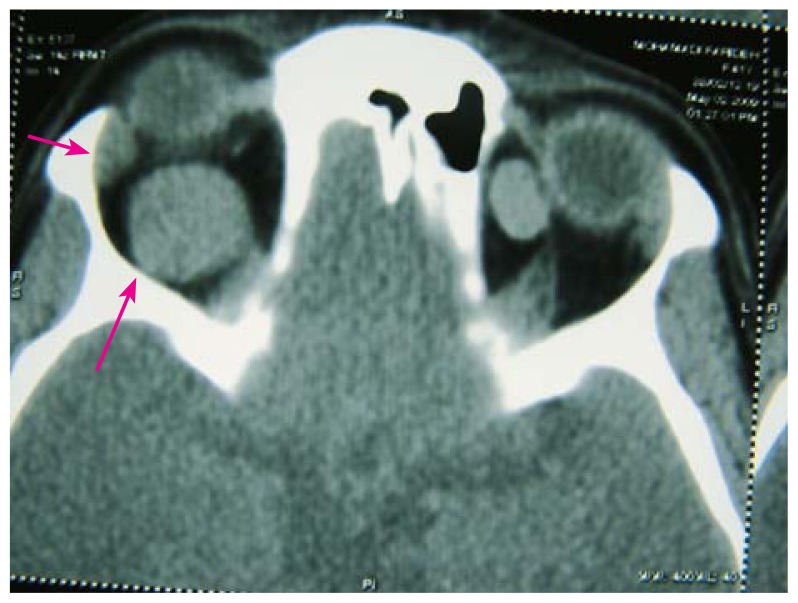
Orbital computed tomography revealed a well-defined round homogenous intraconal soft tissue mass with severe proptosis in the right orbit and a smaller lesion in the superomedial extraconal space of the left orbit.

**Figure 3:**
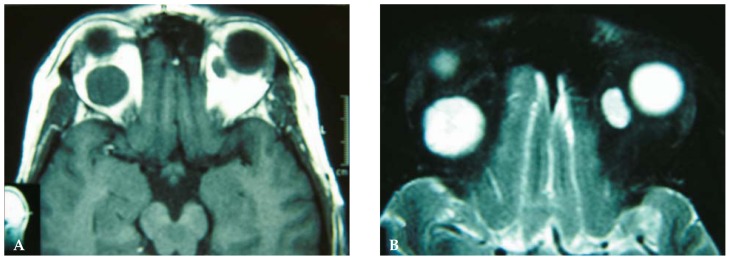
**(A)** T1-weighted magnetic resonance imaging shows a well-defined mass of intermediate density relative to brain tissue and isointense relative to muscle in the orbital cavity on both sides. **(B)** T2-weighted image after gadolinium injection shows marked uniform enhancement of the masses.

**Figure 4:**
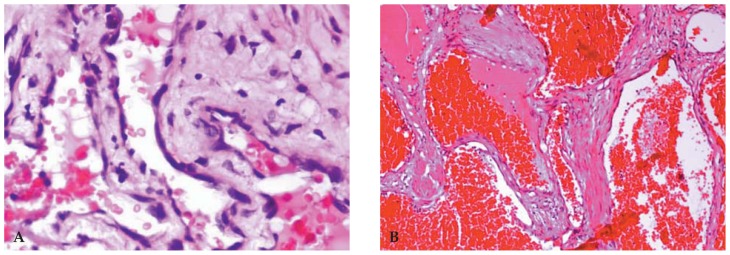
Histopathologic examination (Hematoxylin & Eosin) revealed a well-defined encapsulated mass composed of multiple dilated vascular structures with a single layer of endothelial cell lining (**A**, ×400) containing blood and surrounded by inflammatory fibroconnective tissue (**B**, ×40), findings compatible with cavernous hemangioma.
